# A Complicated Course of Acute Viral Induced Pharyngitis, Icteric Hepatitis, Acalculous Cholecystitis, and Skin Rash

**DOI:** 10.1155/2016/6796094

**Published:** 2016-10-26

**Authors:** Fereshte Sheybani, HamidReza Naderi, Seddigheh Sadat Erfani, Masoumeh Gharib

**Affiliations:** ^1^Department of Infectious Diseases, Faculty of Medicine, Mashhad University of Medical Sciences, Mashhad, Iran; ^2^Department of Pathology, Faculty of Medicine, Mashhad University of Medical Sciences, Mashhad, Iran

## Abstract

This case reveals the complexities and challenges in the diagnosis of acute Epstein-Barr virus (EBV) infection, indicating the potential relationship between EBV infection and severe icteric hepatitis, acalculous cholecystitis, and lymphocytic vasculitis. We suggest including EBV infectious mononucleosis in the list of differential diagnoses when any of these clinical syndromes (or a combination thereof) occurs without apparent cause, especially in the presence of lymphocytosis. To our knowledge, this is the first report to suggest the possible role of EBV in the pathogenesis of cutaneous lymphocytic vasculitis. Also it is possible that EBV infection triggered the flare-up of the underlying rheumatologic disease. Therefore, it could be assumed that a part of the clinical syndrome (e.g., dermatologic manifestations) might be related to the flare-up of the underlying rheumatologic disease.

## 1. Introduction

Infectious mononucleosis is a clinical syndrome characterized by pharyngitis, fever, lymphadenopathy, and presence of atypical lymphocytes in the peripheral blood smear. Infection with Epstein-Barr virus (EBV) is the most common cause of this syndrome [1]. EBV infectious mononucleosis is a common cause of viral pharyngitis in all ages, but it is particularly frequent among young adults. Epidemiologic studies show EBV infection in the majority of adults worldwide. Half the population in industrialized countries is infected in early childhood and most of the rest get infected between 10 and 20 years. However, most of the infection in developing countries occurs in early childhood [2].

Although EBV-associated infectious mononucleosis is a well-known syndrome presenting a wide range of symptoms and signs, it sometimes has an atypical and misleading clinical presentation that leads to diagnostic challenges and dilemma. Acute phase of infectious mononucleosis usually subsides without any sequel within weeks [3]. However, disabling and prolonged illness are also noted [3]. Here, we present a case with a complex and challenging picture of acute EBV infection.

## 2. Case Description

A 23-year-old married woman with two children, a resident of Mashhad in northeastern Iran, was admitted to the Department of Infectious Diseases at Imam Reza Hospital with the complaint of sore throat and generalized skin lesions. Six months prior to admission, she was referred to a rheumatologist because of pain and swelling of the knees, hips, and heels that gradually spread to the small joints of the upper limb. She was treated with ibuprofen and sulfasalazine three weeks before the onset of new symptoms based on the diagnosis of seronegative spondyloarthropathy (i.e., joint disease of the vertebral column with an increased incidence of HLA-B27, as well as negative rheumatoid factor and antinuclear antibody (ANA)). Two weeks after initiation of the prescribed drugs, she developed fever and sore throat and based on finding exudate on the tonsils, streptococcal pharyngitis was diagnosed in an outpatient visit by a general practitioner and intramuscular penicillin G and cefixime were prescribed for three days. She subsequently developed generalized itchy rash and hives, nausea, vomiting, and epigastric pain. The patient was referred back to her rheumatologist who stopped all medications and referred her to hospital.

At the time of admission to the emergency department, she was conscious, oriented, and without any distress but had fever. Vital signs were as follows: blood pressure 117/89 mmHg; heart rate 112 beats per minute; respiratory rate 34 breaths per minute; and body temperature 38.4°C. Nonblanchable erythematous papular rash was evident predominantly over the face, trunk, and proximal and distal limbs including palms and soles, concentrating in the corrugated and pressure areas of the body, and in some areas as erythematous to violet patches. Head and neck examination revealed pharynx and soft palate erythema with bilateral white exudate on the tonsils, bilateral cervical lymphadenopathy in the posterior triangle, and bilateral axillary lymphadenopathy. Other examinations were unremarkable except for mild epigastric tenderness. According to the clinical presentation, the following differential diagnoses were proposed: drug reaction, infectious mononucleosis, systemic lupus erythematosus (SLE), adult-onset still's disease (AOSD), and lymphoproliferative disorders. The results of laboratory tests on admission and during the course of hospitalization can be seen in Table 1. Peripheral blood smear examination revealed 20% (4400) polymorphonuclear cells, 62% (13640) lymphocytes, 3% (660) monocytes, 2% (440) eosinophils, and 13% (2860) atypical lymphocytes. Urine analysis test was normal. Blood culture samples were sent to the laboratory and supportive therapies, including IV fluids, antipyretics, and antipruritic drugs, were started. Blood cultures, monospot test (a form of the heterophile antibody test for infectious mononucleosis due to Epstein-Barr virus), and serology for hepatitis viruses were all negative. It is unclear why the monospot test was negative. However, the sensitivity of the test is only 85% in adults. Abdominopelvic ultrasonography showed hepatomegaly, splenomegaly, and multiple para-aortic lymph nodes. Chest X-ray and echocardiography were unremarkable.

During the first few days of hospitalization, the patient continued to have fever, nausea, and epigastric pain, but upper endoscopy findings were unremarkable. In the meantime, skin lesions increased in number and density and their colors and pruritus intensified; hence skin biopsy was performed for histopathological evaluation.

**Figure 1:**
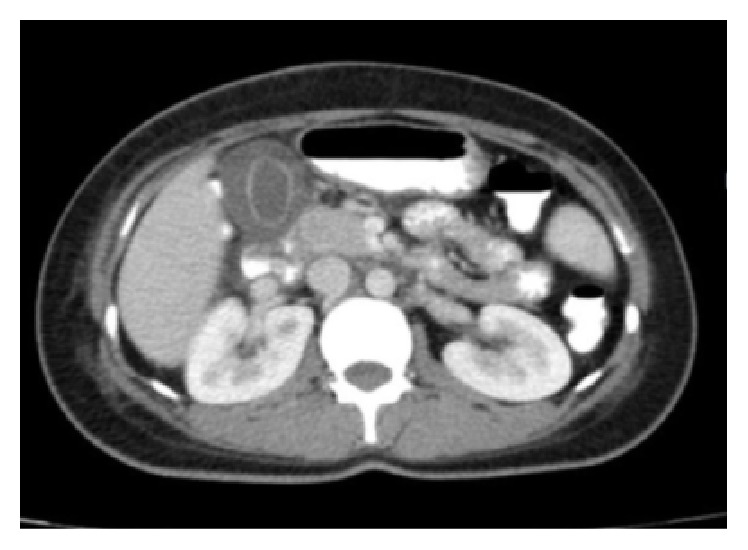
Abdominal CT scan representing an acalculous gallbladder with a thick wall.

On the sixth day of hospitalization, the patient developed severe, more intense epigastric and right upper quadrant pain which was accompanied by nausea and vomiting and positive Murphy's sign. The patient developed jaundice the next day, which increased in intensity along with other symptoms. Moreover, the skin lesions progressed, and mucositis and oral ulcers developed. New laboratory tests showed a rapid increase in liver enzymes and bilirubin level (Table 1). Activated partial thromboplastin time, prothrombin time/international normalized ratio, and amylase and lipase levels were within normal limits. Abdominopelvic computed tomography (CT) scan with oral and intravenous contrast and color Doppler sonography of the hepatic artery were performed, which revealed hepatosplenomegaly, multiple para-aortic and inguinal lymph nodes, pelvic free fluid, and a distended gallbladder with thickened wall, without any stones, suggesting acalculous cholecystitis (Figure 1).

**Figure 2:**
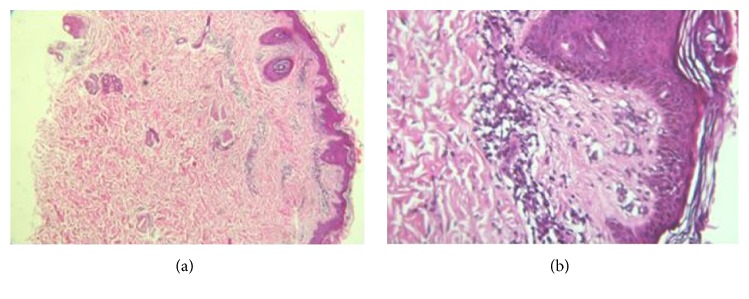
Lymphocytic vasculitis: histopathological examination shows predominantly lymphocytic infiltrate surrounding the walls of small vessels in the dermis accompanied by plumping of endothelial cells ((a), ×40, and (b), ×400, H-E stain).

Serum copper and ceruloplasmin levels, iron profile, and serum protein electrophoresis were within normal limits. Laboratory tests for autoimmune hepatitis, including ANA, ASMA, anti-LKM1, and serum IgG4 were done, which yielded negative results. P-ANCA and C-ANCA were negative, and C3, C4, and CH50 were in normal range. Peripheral blood flow cytometry showed an increase in CD8 T lymphocytes percent up to 68% (normal range: 17–31%) and anti-HLA DR up to 60% (normal range: 7–20%). Histopathological examination of skin biopsy revealed lymphocytic vasculitis (Figure 2) and the results of IgG and IgM antiviral capsid antigen (VCA) and IgG anticytomegalovirus (CMV) were reported positive but IgM anti-CMV was negative. IgG anti-EBNA was not tested due to lack of resources. Due to the possibility of EBV mononucleosis syndrome presenting with skin rash and hepatitis, prednisolone (1 mg/kg per day) was prescribed. The next day, the fever and rash subsided, and liver enzymes gradually decreased. Subsequently, other laboratory test results returned to normal range.

Five days later, patient was discharged with the final diagnosis of EBV hepatitis and acalculous cholecystitis. Patient had no family history of severe EBV or IM like infections. Unfortunately, genetic testing could not be performed on the patient to search for the possible underlying susceptibility to EBV (e.g., mutations in SLAM-associated protein) due to lack of resources. Moreover, further testing for EBV-associated hemophagocytic-like syndromes has not been performed but would be warranted. It could be assumed that skin rash and hepatitis were secondary to the underlying connective tissue disease, drug reaction, or acute viral syndrome. However, based on the clinical syndrome of fever, exudative pharyngitis, and lymphocytosis, accompanied by positive EBV serology results, the diagnosis of a severe form of EBV infectious mononucleosis complicated by icteric hepatitis, skin vasculitis, and acalculous cholecystitis was considered. Prednisolone was tapered over 3 weeks. However, low dose of prednisolone (7.5 mg per day) was continued for the treatment of underlying spondyloarthropathy. After a 6-month clinical follow-up, the patient was symptom-free and the follow-up laboratory tests remained within normal limits.

## 3. Other Similar and Contrasting Cases in the Literature and Discussion

The present case is an example of diagnostic challenges and complexities in a patient with EBV-associated infectious mononucleosis complicated by acalculous cholecystitis who simultaneously developed icteric hepatitis, skin rash, and mucosal involvement. Although it was not proved that the latter two were also EBV-induced, the concurrency suggests a potential role of the virus in the whole scenario. It seems also likely that EBV infection triggered the flare-up of the underlying rheumatologic disease.

The classic clinical presentation of infectious mononucleosis in adolescents or in adulthood is fever, oropharyngitis, and bilateral lymphadenitis. Hematologic findings consistent with infectious mononucleosis include a differential that demonstrates greater than 50% lymphocytes, an absolute lymphocyte count greater than 4500, or an elevated lymphocyte count with greater than 10% atypical lymphocytes. Liver function tests are often abnormal and raised liver enzymes in a patient with pharyngitis are strongly in favor of the diagnosis of infectious mononucleosis. Other laboratory tests include leukocytosis, with occasional relative neutropenia and self-limiting thrombocytopenia [1, 4]. Although heterophile antibody test is not sensitive, the positive result can reject the need for further investigation. EBV specific antibodies are used in patients with suspected EBV infectious mononucleosis with negative heterophile antibody test. The diagnosis is often made clinically [4]. The presence of IgM anti-VCA is strongly associated with acute infection and its level remains positive for 4 to 8 weeks [1].

The occurrence of acute acalculous cholecystitis during the course of EBV infection is rare but reported, especially in those patients with cholestatic hepatitis [5, 6]. Although cholecystitis is not a common manifestation of primary EBV infection and hepatobiliary ultrasound is not done routinely, the elevated level of alkaline phosphatase may indicate a cholestatic pattern and suggest the need for further evaluation [7]. Therefore there are some who suggest performing a biliary ultrasound examination in a patient with primary EBV infection who complains of epigastric pain [8]. Acute acalculous cholecystitis typically occurs as a secondary event in patients who are hospitalized, are acutely ill with another disease, and need to be treated with antibiotics and, in some cases, surgical intervention. However, a survey of the literature shows that, unlike other causes of acute acalculous cholecystitis, these treatments do not seem to be useful in acute acalculous cholecystitis associated with EBV infection [9]. Hence, it has been pointed out that acute EBV infection should be considered as a possible etiological factor of acalculous cholecystitis [10], especially among young people, because the vast majority of these cases do not require antibiotic treatment and surgery [7]. With the exception of a 6-year-old child, all eight cases reported so far were young women (ages 17 to 34), who were treated with supportive care and follow-up, without any need for surgical intervention. It has been proposed that, in similar cases, surgery should be considered only when patient's condition deteriorates or ultrasound criteria indicate the need for surgery [5].

There were several other rare aspects in the patient presented in this case report. She was suffering from icteric hepatitis, with a serum bilirubin level greater than 10 mg/dL and transaminases higher than 1000 U/L, who had a progressive course during the first week of hospitalization that eventually subsided with the administration of corticosteroids. EBV hepatitis is generally known as a complication of infectious mononucleosis. However, EBV hepatitis and cholestatic jaundice have been recently described in patients without the clinical manifestations of EBV-associated infectious mononucleosis. Although abnormal liver function tests occurs in approximately 80% of patients with EBV infection, symptomatic hepatitis is rare and EBV hepatitis is usually not diagnosed clinically. Compared with infectious mononucleosis, EBV hepatitis affects an older age group, so that half of reported patients were older than 60 years [11]. In a study by Vine et al. [11], only 2 out of 17 patients with EBV hepatitis showed the manifestations of infectious mononucleosis, both of whom were young, that is, the most common age group being affected by infectious mononucleosis. EBV hepatitis is generally characterized by nonspecific symptoms such as anorexia, weight loss, abdominal pain, nausea, vomiting, and flu-like symptoms. Unlike other forms of acute viral hepatitis (e.g., hepatitis A, B, and E), EBV hepatitis is rarely associated with alanine aminotransferase (ALT) greater than 1000 U/L and is usually accompanied by lymphocytosis. EBV hepatitis usually has a cholestatic pattern, so that anicteric cholestatic hepatitis is observed in approximately 60% of the patients, while jaundice occurs only in 5–7% of all patients [11, 12]. The simultaneous presence of lymphocytosis and splenomegaly, which is seen in the vast majority of patients, should raise the suspicion for EBV. However, these patients have moderate enlargement of the spleen that can only be diagnosed by ultrasound. Therefore, the diagnosis of EBV hepatitis should be considered in all patients with unexplained hepatitis irrespective of their age, especially when fever and anicteric cholestatic pattern occur simultaneously, and should be followed by routine serological tests [11, 12]. Although EBV hepatitis is usually self-limiting and recovers fully after a few weeks, a small number of patients show symptoms severe enough to require hospitalization [11]. Fulminant EBV hepatitis has been also described and liver failure typically occurs in immunocompromised patients. In some cases, fulminant EBV hepatitis has been reported to be treated favorably with antiviral drugs such as ganciclovir and valganciclovir [13]. However, the pathogenesis of infectious mononucleosis, including hepatic involvement, has been suggested to be essentially immune-mediated. Indeed, various studies on the pathogenesis of EBV-associated liver disease have shown that liver damage occurs through the mediation of EBV-infected T lymphocytes through soluble products of immune response. In these studies, the severity of the damage has also been shown to be associated with high viral burden and, consequently, the level of the infected CD8 T lymphocytes [13].

A generalized rash which can present as maculopapular, petechial, or urticarial rash may occur in patients with infectious mononucleosis, especially following the administration of ampicillin. Although the underlying mechanism is poorly understood, it does not seem to be related to drug allergies [4]. Skin lesions in the presented case were pathologically diagnosed as lymphocytic vasculitis. Lymphocytic vasculitis is a group term for a number of clinically heterogeneous diseases which on histopathological examination show lymphocytic vasculitis. This syndrome is assumed to be associated with an aberrant hypersensitivity reaction to an antigen such as an infectious agent, a drug, an endogenous antigen, or foreign bodies. Lymphocytic vasculitis is nonspecific and can be seen mainly in connective tissue disorders (particularly SLE) and less commonly in various other diseases, including infections. In the English literature, there are few reports on the incidence of lymphocytic vasculitis associated with hepatitis A vaccine, anthrax, hepatitis B virus infection and vaccination, and flu vaccine [14–17]. A review of the published articles on the relationship between EBV and lymphocytic vasculitis associates this pathology with X-linked lymphoproliferative diseases related to ongoing or prior EBV infection, particularly in the nervous tissue [18, 19]. The formation of this form of vasculitis has been attributed to the impaired immunologic response to EBV, which occurs as a result of functional inactivation of SLAM-associated protein (SAP) secondary to SAP mutations, which leads to EBV infection of vascular structures [18, 19]. However, no relationship was found between EBV and cutaneous lymphocytic vasculitis. This report suggests a possible etiologic role of EBV in the cutaneous lymphocytic vasculitis in the presented patient. However, as the vascular infiltrate was not immunophenotyped and EBV DNA PCR was not performed, the causal role of EBV in the development of this patient's skin lesions remains uncertain.

## Figures and Tables

**Table 1:** Laboratory test results on admission and during the course of hospitalization.

Day of admission	1st	3rd	5th	7th	9th	11th	15th	17th
White blood cells (/mm^3^)	22000		8300	6600		7200	7800	5700
Polymorphonuclear leukocytes (count)	4400		3403	3009		1872	3556	2280
Lymphocytes (count)	13640		3320	3128		4752	3517	2661
Hematocrit	41		37	30.2		32.2	32.3	32.5
Platelets (/mm^3^)	194000		133000	113000		104000	124000	238000
Aspartate aminotransferase (U/L)	169	396	592	1228	442	64	40	27
Alanine aminotransferase (U/L)	641	541	616	1538	1118	503	214	176
Alkaline phosphatase (U/L)	909	1209	1753	1695	1269		996	949
Gamma-glutamyl transferase (U/L)				212	263	167		
Total bilirubin (mg/dL)	2.3	2.3		11.9	11.9	4.1		2.3
Direct bilirubin (mg/dL)	1.1	1.3		6.9	6.2	2.4		1.4
Erythrocyte sedimentation rate (mm/h)	8							
